# Supplementary figures and images for: The impact of malaria-induced neutrophil subset shift and a link to Burkitt lymphoma
Source: PLoS One. 2026 Jun 1;21(6):e0348729. doi: 10.1371/journal.pone.0348729 (PMC13225646; doi:10.1371/journal.pone.0348729)

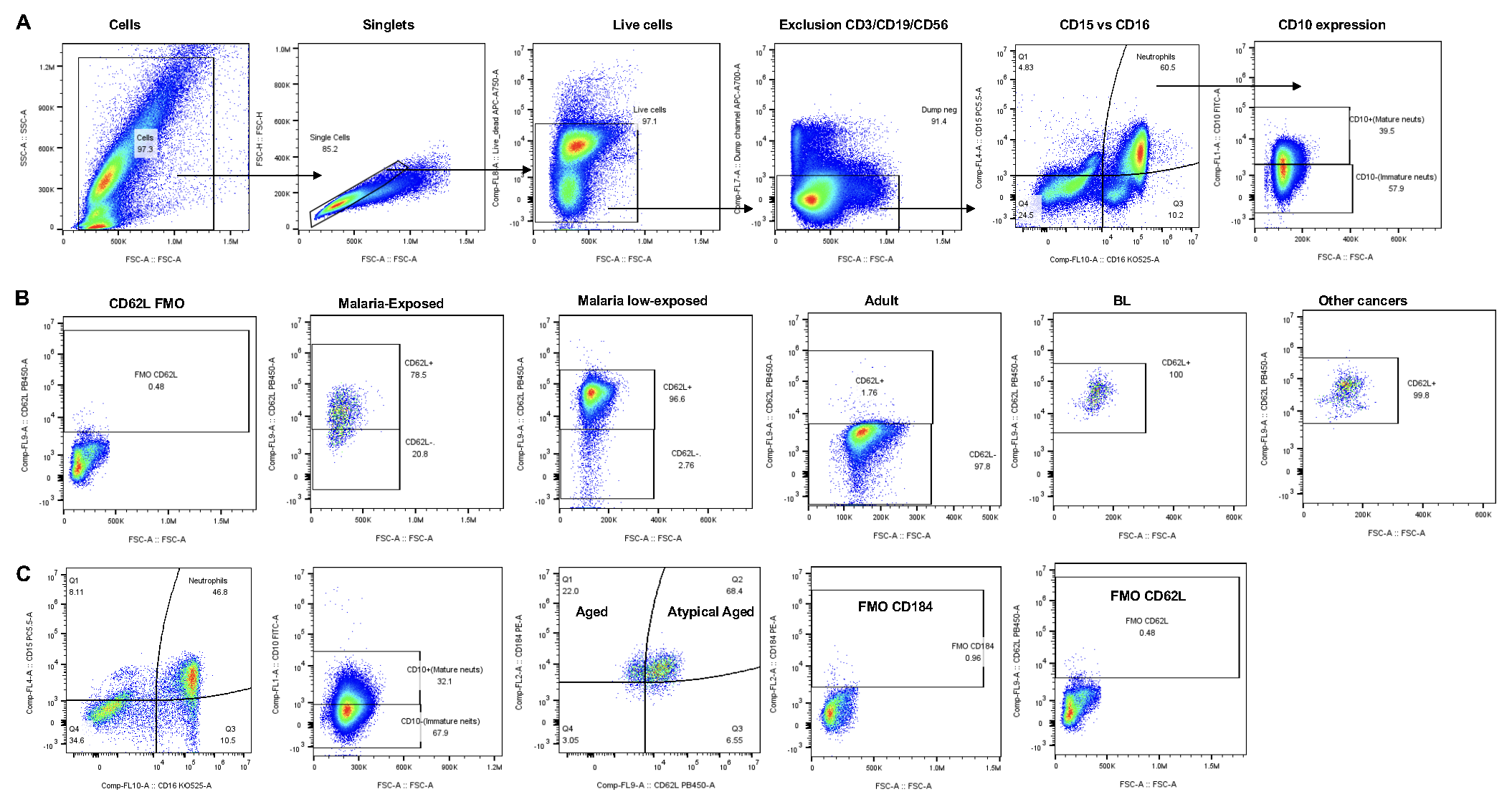

Supplement: S1 Fig — (A) Flow cytometry gating strategy used to phenotype neutrophils. (B) CD62L gating based on FMO for malaria-exposed and malaria low-exposed children, adults, children with BL or other cancer. (C) Gating strategy used to identify atypical aged neutrophil subset. (TIFF) [file pone.0348729.s001.tiff]

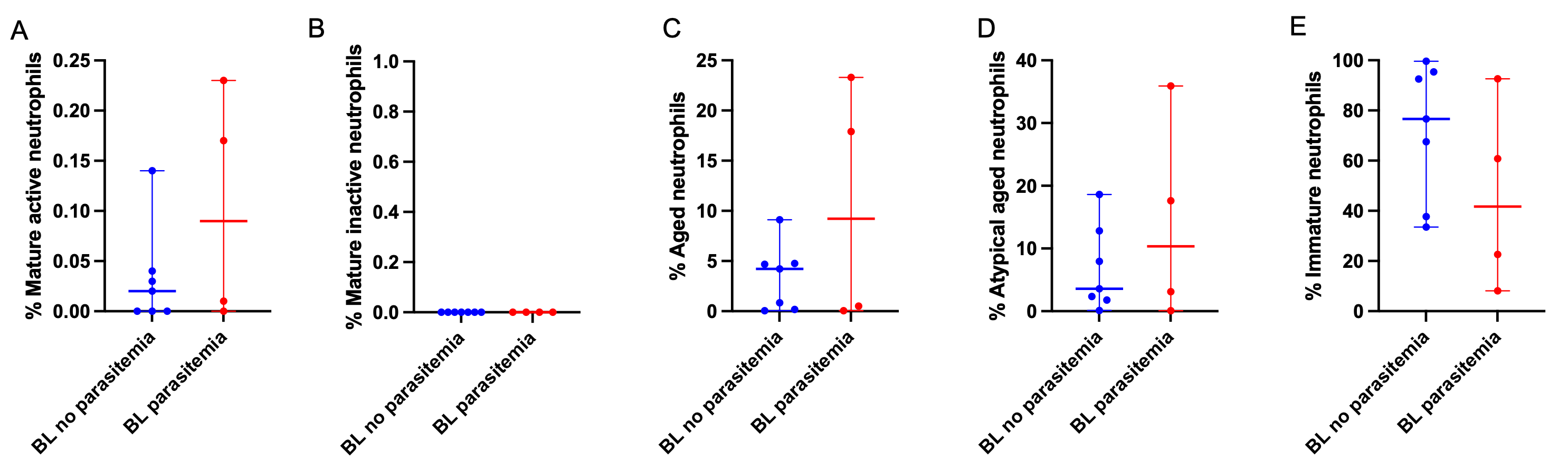

Supplement: S2 Fig — (TIFF) [file pone.0348729.s002.tiff]

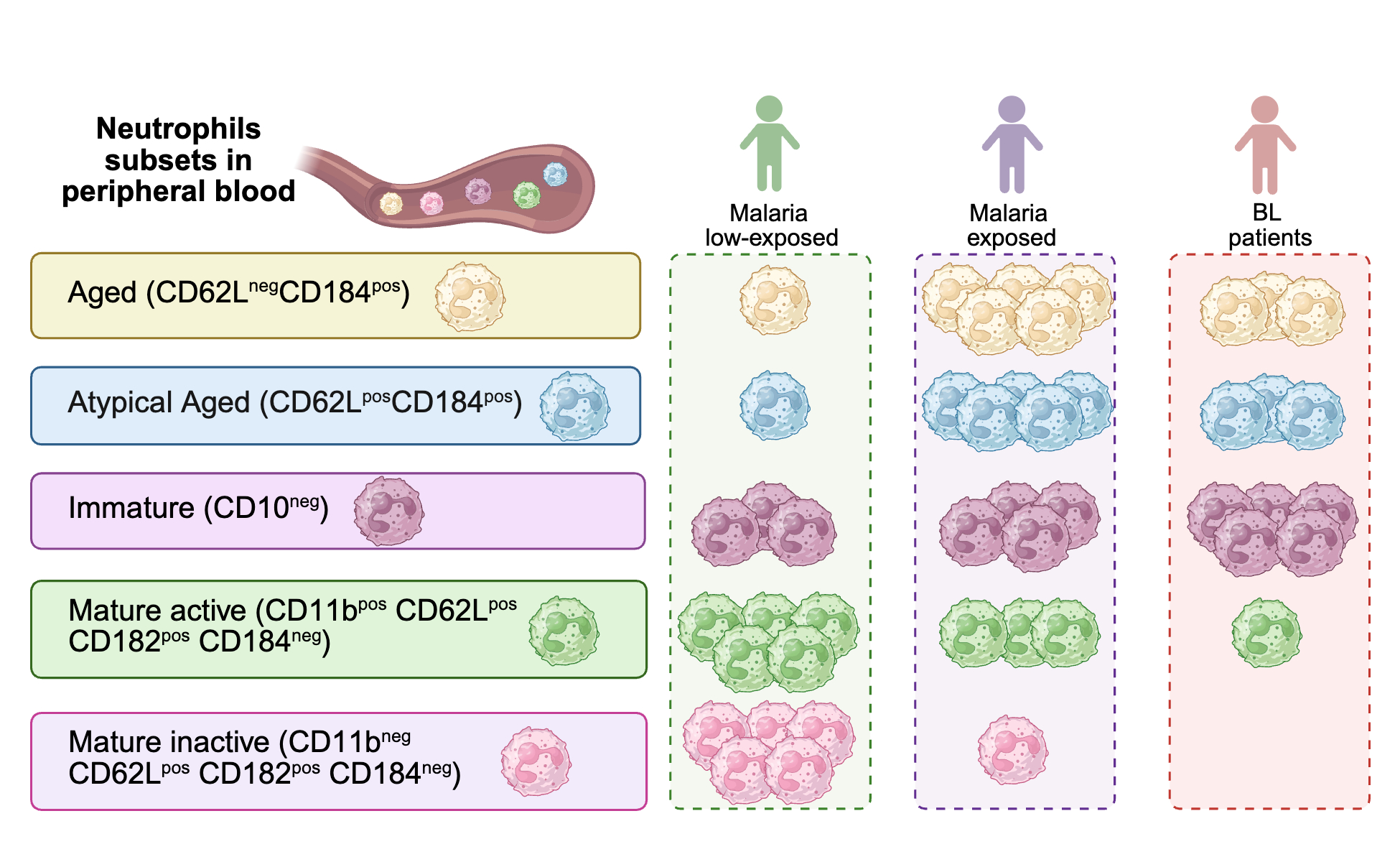

Supplement: S4 File — (JPEG) [file pone.0348729.s009.jpeg]
