# Supplementary material for: The impact of malaria-induced neutrophil subset shift and a link to Burkitt lymphoma
Source: PLoS One. 2026 Jun 1;21(6):e0348729. doi: 10.1371/journal.pone.0348729 (PMC13225646; doi:10.1371/journal.pone.0348729)
Supplement: S1 Table — (DOCX) [file pone.0348729.s003.docx]

**S1 Table: Neutrophil flow cytometry antibody panel (Biolegend)**

| **Antibody** | **Fluorochrome** | **Clone** | **RRID** |
| --- | --- | --- | --- |
| CD3 | Alexa Fluor700 | OKT3 | AB_2563408 |
| CD19 | Alexa Fluor700 | HIB19 | AB_2616936 |
| CD56 | Alexa Fluor700 | 5.1H11 | AB_2564099 |
| CD15 | PerCP | HI98 | AB_893256 |
| CD16 | BV510 | 3G8 | AB_2562085 |
| CD10 | FITC | HI10a | AB_314919 |
| CD11b | BV605 | ICRF44 | AB_256202 |
| CD62L | BV421 | DREG-56 | AB_2562914 |
| CD182 | APC | 5E8-C7-F10 | AB_492936 |
| CD184 | PE | 12G5 | AB_314612 |
