# Supplementary material for: The impact of malaria-induced neutrophil subset shift and a link to Burkitt lymphoma
Source: PLoS One. 2026 Jun 1;21(6):e0348729. doi: 10.1371/journal.pone.0348729 (PMC13225646; doi:10.1371/journal.pone.0348729)
Supplement: S2 Table — (DOCX) [file pone.0348729.s004.docx]

**Supp. table 2: Demographics and characteristics of the children participants.**

^A^: Median [min-max]*,* ^P^: *p*-value from the Mann-Whitney statistical test, ^C^: *p*-value from the Chi-square statistical test, #: number of individuals, NT: Not Tested.

| Demographics | Malaria exposed (ME, n=19) | Malaria low-exposed  (MLE, n=11) | BL Cases   (n=11) | Other Cancer Cases  (OC, n = 5) | ME vs. MLE | ME vs. BL | MLE vs. BL | BL vs. OC |
| --- | --- | --- | --- | --- | --- | --- | --- | --- |
| Age^A^ (years) | 5  [4-7] | 5  [4-7] | 6  [3-9] | 8  [6-14] | 0.71^P^ | **0.03**^P^ | 0.23^P^ | 0.07^P^ |
| Sex  (% of males) | 52.6% | 36.3% | 81% | 60% | 0.40^C^ | 0.11^C^ | **0.03**^C^ | 0.37^C^ |
| ANC^A^  (10^3^/µL) | 32.35  [18-69] | 40.2  [26.1-60.5] | 58.4  [37.6-76.2] | 74.75  [60.5-87.5] | 0.09^P^ | **0.0001**^P^ | **0.02**^P^ | **0.04**^P^ |
| WBC^A^ (10^3^/µL) | 6.1  [3.1-9.2] | 7.22  [5.48-8.28] | 8.26  [5.1-13.28] | 6.49  [4.12-8.15] | 0.05^P^ | **0.01**^P^ | **0.03**^P^ | 0.18^P^ |
| qPCR parasitemia % pos. (#)  Median [range] | 68% (13/19)  13 [0-30,517.40] | 0% (0/11)  0 [0-0] | 36% (4/11)  0 [0-5] | 20% (1/5)  0 [0-0.09] | **0.0001** | **0.001** | 0.11 | 0.88 |
| qPCR EBV load  % pos. (#)  Median [range] | NT | NT | 100% (11/11)  8,952.85 [2.95-1,651,986.82] | 100% (5/5)  219.02 [32.66-867.28] | NT | NT | NT | 0.01 |
| Hemoglobin (g/dL)^A^ | 12.3 [10.6-14.1] | 13.7 [11.8-15.7] | 8.7 [7-17] | 9.2 [8-14] | **0.01**^P^ | **0.002**^P^ | 0.006^P^ | 0.70^P^ |
| AMA1 (MFI)^A^ | 72,126 [384-217,702] | 0 [0-2,541] | 2495 [0-171,811] | 197 [0-153,896] | **0.0001**^P^ | 0.10^P^ | **0.005**^P^ | 0.27^P^ |
