# Supplementary material for: The impact of malaria-induced neutrophil subset shift and a link to Burkitt lymphoma
Source: PLoS One. 2026 Jun 1;21(6):e0348729. doi: 10.1371/journal.pone.0348729 (PMC13225646; doi:10.1371/journal.pone.0348729)
Supplement: S3 Table — (DOCX) [file pone.0348729.s005.docx]

**Supp. table 3**: **Demographics and characteristics of the adult participants.** ^A^: Median [min-max], ^P^: *p*-value from the Mann-Whitney statistical test, ^C^: *p*-value from the Chi-square statistical test, #: number of individuals.

| Demographics | Age^A^  (years) | Sex  (% of males) | ANC^A^  (10^3^/µL) | WBC^A^  (10^3^/µL) | Hgb^A^  (g/dL) | AMA1^A^  (MFI) | qPCR parasitemia % pos. (#)  Median [range] |
| --- | --- | --- | --- | --- | --- | --- | --- |
| Healthy Adults  (n=3) | 29 [26-34] | 100% | 52.9 [35.9-66.2] | 5.24 [5.13-5.712] | 16 [15.5-16] | 31,108 [104-5,977] | 0% (0/3)  0 [0-0] |
