# Supplementary material for: The impact of malaria-induced neutrophil subset shift and a link to Burkitt lymphoma
Source: PLoS One. 2026 Jun 1;21(6):e0348729. doi: 10.1371/journal.pone.0348729 (PMC13225646; doi:10.1371/journal.pone.0348729)
Supplement: Attachment — Submitted filename: Comments.docx [file pone.0348729.s010.docx]

The manuscript titled ‘The Impact of Malaria-Induced Neutrophil Subset Shift and a Link to Burkitt Lymphoma’ presents a strong and interesting study, but the following concerns should be addressed before publication.

Major:

1. The authors should clarify the difference between ‘mature’ and ‘mature inactive’ neutrophils, as both populations appear to share the same surface marker expression in the Discussion (lines 286–287) and in the legends of Figures 1 and 3.
2. Neutrophil subsets are defined using surface marker expression; however, these markers do not explain functional heterogeneity or suppressive activity. Consequently, the immunosuppressive or tumor-promoting roles linked to the expanded immature and aged neutrophil subsets remain speculative.
3. Statements made in lines 311-313 should be supported by the corresponding dataset
4. For lines 149-151, the authors should provide the Ct values and define the thresholds and criteria used for downstream parasite quantification. Also add the primer seq in supplementary material.
5. The authors should clarify whether malaria-exposed children (n = 19) were defined by residence or by lab data, and specify the proportion with active, treated, and asymptomatic malaria at the time of sampling.
6. The authors infer that a malaria-driven expansion of immature neutrophils contributes to BL immunopathology; however, no supporting BL-specific data are provided. Inclusion of BL-associated protein or transcriptional markers would substantiate this link.

Minor:

a. The authors should include the ethics approval ID numbers for all approving institutes.
b. Sentence referring to the graphical abstract should be removed from lines 215-216.
c. Figure S1 resolution should be improved, and need to include all relevant dot plot data to the Supplementary Material.
