# Supplementary material for: The impact of malaria-induced neutrophil subset shift and a link to Burkitt lymphoma
Source: PLoS One. 2026 Jun 1;21(6):e0348729. doi: 10.1371/journal.pone.0348729 (PMC13225646; doi:10.1371/journal.pone.0348729)
Supplement: Attachment — Submitted filename: Response to reviewers.docx [file pone.0348729.s012.docx]

**Response to the reviewers.**

We thank the reviewers and the editorial team for their time and feedback on our manuscript “The impact of malaria-induced neutrophil subset shift and a link to Burkitt lymphoma” (PONE-D-25-56348). The feedback has significantly improved the clarity, rigor and interpretation of our study’s results. In response, we have substantially revised the manuscript, adding data on *Pf*-malaria parasitemia (tables S2 and S3), EBV load (table S2), and a supplemental figure (supplemental figure 2) showing neutrophil subset profiles among BL participants with and without parasitemia. We edited our figures to match the new definition of our malaria low-exposed healthy children group (MLE) and we added “active” to the “mature neutrophil plots” to better differentiate them from the mature inactive neutrophils. We have also adjusted the tone of our manuscript to focus on hypothesis-driven results throughout the manuscript. The lines indicated in the responses below are based on the manuscript with the track change to allow the reviewer to evaluate the response to their comment.

We sincerely thank the reviewers for their constructive suggestions, which have informed substantial improvements in the study’s methodological soundness and significance.

**Reviewer #1**: Major comments
1. Individuals residing in malaria-prone areas whether seasonal, epidemic-prone, or endemic cannot be appropriately classified as malaria-naïve. The presence of positive malaria results (see S2 Table) further invalidates the use of the term “malaria-naïve” for this group. **The authors should therefore adopt an alternative and more accurate terminology to describe these participants.**

*Response #1*: We thank the reviewer for this and the following comments. We changed “malaria-naïve” to “malaria low-exposed” throughout the manuscript to acknowledge that our reference group nevertheless resides in a malaria endemic country.

2. The term **“healthy group” should be explicitly defined in** the Methods section to avoid ambiguity for example, whether “healthy” refers to the absence of cancer, malaria infection, or other clinical conditions.

*Response #2*: To avoid ambiguity, the healthy group has been defined in the Methods section, lines 110-111 of the revised manuscript as follows: “from healthy children (cancer free, overall good health and without clinical symptoms of malaria such as fever for at least 48 hours prior sample collection)...”.

3. Include the **number of malaria-positive cases by PCR in malaria naïve participants and the 3 adults in text within the result section**.

*Response #3*: We have included these values in the revised manuscript, in the results section under the demographics, lines 237 as follows, “none of the malaria low-exposed or adults had malaria parasitemia by qPCR”.

4. The criteria used to select the three adult participants are unclear. The authors should justify **why only three adults were included** and explain why this number differs from the fixed number of children selected.

*Response #4*: We initially intended to include a larger number of healthy adult participants. However, for downstream analysis, only samples that passed our QC which includes: quality and integrity of the samples (samples reaching the laboratory within 2 hours of blood draw were considered eligible for consistency in processing); quality of the flow cytometry data (a minimum of 450,000 cells acquired, a minimum of 95% of viability of these cells). Among the adult samples collected, only three met this predefined criterion and were therefore included in the final analysis. We have clarified this quality control criterion in the Methods section, Lines 116-119 of the revised manuscript as follows: “Based on our quality control criterions which includes quality and integrity of the samples (samples reaching the laboratory within 2 hours of blood draw were considered eligible for consistency in processing); quality of the flow cytometry data (a minimum of 450,000 cells acquired, a minimum of 98% of viability of these cells), only 3 adults were retained for the downstream analysis.”

We recognize this is a limitation of the study but due to these logistical hurdles, financial constraints and timeline to completion of study, we were not able to collect anymore blood samples from adults. Adult samples were meant to be internal controls for our assay, not our primary goal which was comparison between groups of children. However, we do not believe this changes the interpretation of our findings. (See additional responses and editorial changes below).

5. Given the very small number of adults included (n = 3), the **data are insufficient to support the conclusions drawn from this group….**“Malaria-exposed children also had neutrophil profiles that closely resembled those seen in the adults.” Also, among the adult samples, AMA1A (MFI) was 31,108 [104-5977], indicating prior interaction with Plasmodium. If this were detected in one of the three adult samples, it could represent a substantial proportion relative to the small sample size and may differ considerably from observations made in larger cohorts, thus misleading.

*Response #5*: We agree with the reviewer. The small number of adults included does not allow a robust interpretation and so we have excluded this sentence from our abstract (line 34). We also changed the title of our result to “Cumulative exposure to malaria during childhood leads to reduced abundance of mature active and inactive neutrophils, reaching levels similar to those observed in adults” line 304-305. Finally, in the discussion (line 347-348), we changed the sentence as follows: “ This shift in neutrophil subsets was also observed in adults, however, chronic malaria exposure drives a shift towards an atypical neutrophil phenotype in malaria-exposed children whereas, as expected, conventional aged neutrophils were the most abundant in adults”.

To make sure we did not have a bias due to asymptomatic malaria infection, we assessed *Pf*-malaria parasitemia and none of the adults had current infection.

**Reviewer #2**: The manuscript titled ‘The Impact of Malaria-Induced Neutrophil Subset Shift and a Link to Burkitt Lymphoma’ presents a strong and interesting study, but the following concerns should be addressed before publication.

Major:
1. The authors should clarify the **difference between ‘mature’ and ‘mature inactive’ neutrophils**, as both populations appear to share the same surface marker expression in the Discussion (lines 286–287) and in the legends of Figures 1 and 3.

*Response #6*: We thank the reviewer for this important comment. We have clarified the difference between mature and mature inactive neutrophils by correcting the typo in the surface markers in the legends for figure 1 and 3 and also in the discussion, line 368 of the revised manuscript. We would also like to add that CD11b is the activation marker in neutrophils, therefore, mature neutrophils that do not express CD11b are inactive. We therefore added the word “active” to the mature CD11b positive neutrophil subset throughout the manuscript.

2. Neutrophil subsets are defined using surface marker expression; however, these markers do not explain functional heterogeneity or suppressive activity. Consequently**, the immunosuppressive or tumor-promoting roles linked to the expanded immature and aged neutrophil subsets remain speculative.**

*Response #7*: We agree that surface markers were used to identify neutrophil subsets, but were not used to assess functionality directly. However, we linked the observed expansion of the immature subset in our study to prior literature (Mackey et. al, PMCID: PMC6702268), showing tumor-promoting or immunomodulatory roles of this subset. To address the reviewer’s concern, we have restructured the abstract to ensure the conclusion maintains a suggestive tone and that our interpretation is based on previous literature. This is shown in lines 37-40 of the revised manuscript which state that, “These findings suggest a malaria-associated expansion of the immature neutrophil subset. While functional assays were not performed in this study, previous reports indicate that immature neutrophils can exhibit tumor-promoting functions. Therefore, the observed shift in neutrophil profiles may reflect phenotypic changes associated with malaria exposure that could contribute to a permissive environment for BL. ”

3. **Statements made in lines 311-313 should be clearly supported by the corresponding dataset**

*Response #8*: We agree that the original wording may have implied functional and dynamic interpretations beyond the scope of our cross-sectional phenotypic data. We have revised this section to clarify that our observation is limited to the phenotypic heterogeneity within the aged neutrophil compartment and that further studies are needed to characterize the function and the origin of the atypical aged subset. This is shown in line 384-385 of the revised manuscript as follows, “While our data demonstrate phenotypic heterogeneity within the aged neutrophil compartment, the underlying dynamics and functional properties of these subsets were not assessed.”

4. For lines 149-151, the authors should **provide the Ct values and define the thresholds and criteria** used for downstream parasite quantification. Also, add the primer sequence in the supplementary material.

*Response #9*: The primer and probe sequences have been added in the revised manuscript in line 176-177 as follows, “forward primer (cccatacacaaccaaytgga), reverse primer (ttcgcacatatctctatgtctatct) and probe sequences (6-FAM-trttccataaatggt-NFQ-MGB)”

We used the standard curve analysis criteria for downstream parasite quantification and this has been shown in the revised manuscript line 184-187 as follows, “We applied a standard curve analysis to determine downstream parasite densities using 10-fold serial dilution of 3D7 strain DNA cell as positive control (10,000, 1,000, 100, 10, 1, and 0.1 copies per μL)”. The threshold was set at Ct value of 20 cycles, and this has been shown in the revised manuscript,line 192-195 as “The positivity threshold was set-up at Ct value of 20 cycles”. An Excel file with the Cq values has also been added in the supplementary materials.

5. The authors should clarify **whether malaria-exposed children (n = 19) were defined by residence or by lab data, and specify the proportion with active, treated, and asymptomatic malaria at the time of sampling.**

*Response #10*: In the revised manuscript, we have clarified that the malaria-exposed healthy controls were enrolled by residence and the epidemiology of malaria in these regions, followed by laboratory confirmation by qPCR and serology (lines 114-115) as follows, “The healthy children were enrolled based on the epidemiology of malaria where they reside. Post sampling, current and cumulative malaria exposure was confirmed by qPCR and serology, respectively.”

At the time of blood sampling, 68% of the malaria-exposed children were asymptomatic for malaria. This is shown in the results section in line 236, as “We found that 68% of the malaria-exposed healthy controls had asymptomatic malaria”.

6. The authors infer that a malaria-driven expansion of immature neutrophils contributes to BL immunopathology; however, **no supporting BL-specific data are provided**. Inclusion of BL-associated protein or transcriptional markers would substantiate this link.

*Response #11*: We appreciate the reviewer’s insightful comment. We acknowledge that this study did not examine Burkitt lymphoma-specific molecular or tumor-associated markers. Our primary aim was to investigate neutrophil subset profiles and explore their possible connection to BL pathogenesis, as supported by existing literature such as immature neutrophils found in other types of cancer (Mackey et. al, PMCID: PMC6702268), which we also found highly abundant in our BL patients. Including BL-related proteins or transcriptional markers would exceed the intended scope of this specific study, however, it is included in our next project.

Minor:
a. The authors should include the **ethics approval ID numbers for all approving institutes**.

*Response #12*: We apologize for missing them in the original submission. All Ethic committee numbers have been added to the manuscript lines 127 to 131 as follows: “Written informed consent was obtained from each adult participant and child’s guardian. Ethical approvals were obtained from the Kenya Medical Research Institute (KEMRI) Scientific and Ethical Research Unit (approval number KEMRI/SERU/CGHR/05-06-386/4311), MTRH Institutional Research and Ethics Committee (IREC, approval number 0001911), and the University of Massachusetts Chan Medical School Institutional Review Board (IRB, approval number H00004587).” The Ethics statement paragraph was moved right under the “Participants” section for better clarity.

b. **Sentence referring to the graphical abstract should be removed from lines 215-216**.

*Response #13*: The sentence referring to the graphical abstract has been removed in the revised manuscript line 290.

c. **Figure S1 resolution should be improved** and need to include all relevant dot plot data to the Supplementary Material.

*Response #14*: We have improved Figure S1 resolution to match the journal’s required quality in the supplementary material.

**Reviewer #3**: The authors have presented a well written and detailed account on understanding the shift in neutrophil profiles observed during malaria-induced immunopathy, associated with aggressive Burkitt lymphoma. The data is precise and the manuscript is easy to follow.

*Response #15*: We thank the reviewer for taking the time to review our manuscript and we appreciate the interest in our work.

**Reviewer #4**: Overall: The manuscript’s primary strength is describing malaria-associated neutrophil phenotypes and a novel use-case; however, the extension to a BL pathogenesis ‘bridge’ is insufficiently supported by the presented data and **should be framed as hypothesis-generating or more exploratory,** specification of this might strengthen ties to the conclusions. The methods and gating strategies are clearly elucidated with pertinent details for reproducibility. Detailed critique is below:

Introduction: would benefit from a **clearer explanation of what is known about neutrophils in malaria**, **especially differences between acute infection vs chronic/repeated exposure**, **and why these distinctions matter for interpreting potential links to BL**. The authors should state explicitly what gap their study fills-specially as the paper is being presented as bridging the link between malaria-neutrophils and BL.

*Response #16*: We thank the reviewer for this insightful comment. We have revised the introduction, lines 66-77, to provide a clearer overview of what is currently known about neutrophil responses in malaria. In the revised introduction, we also highlight the limited understanding of how prolonged malaria exposure may shape neutrophil subsets and their potential relevance to BL, and clearly state the gaps and how our study is filling these gaps.

**Definitive wording**: words such as "defects" in the introduction are too strong, precise immunological mechanisms remain incomplete and not fully mapped, **impairment might be a better word to address these.**

*Response #17*: We agree that the word “ defects” in the introduction is too strong and we have replaced it with “impairment” in line 48 and 50 of the revised manuscript

Study design: unclear if the malaria exposure was quantified for BL participants and how it was incorporated into analyses; exposure differences could substantially affect interpretation. The manuscript highlights BL, **was EBV status/viral load (and tumor EBV status where applicable) was assessed**? It may be difficult to distinguish malaria-associated effects from cancer/EBV-driven immune remodelling in this cross-sectional design. Separating BL and non-BL associated effects, malaria vs cancer driven effects and so on might add value to the main interplay the paper wants to address.

*Response #18*: We thank the reviewer for this insightful comment. We performed *Pf* parasitemia on all our BL participants and found that 4 had asymptomatic infection (see revised table 2). We evaluated whether there were any differences in the neutrophil subset profiles between BL participants with and without *Pf* parasitemia and found no statistically significant differences between these two groups, as shown in the plot below (added as supplemental figure 2 in the revised manuscript). Interestingly, the BL participant with the lowest immature neutrophil subset had the highest proportions of aged and atypical aged neutrophils. We further examined the clinical data from these 4 participants and did not find any parameter that distinguished this participant from the other 3. We added this result line 288-289 of the revised manuscript as follows: “Of note, we did not observe any differences in the neutrophil subset profiles between BL participants who had asymptomatic malaria and the ones who did not (Supp. Fig. 2).”

We also measured EBV load from our BL participants and found that all of them had viremia. The median viremia was 8,952.85 with a range of [min: 2.95; max: 1,651,987], which is consistent with endemic BL characteristics observed in this region of Kenya, as we have shown in a previous study (Kaymaz et. al, PMCID: PMC5471630). We added these results in the supplemental table 2. EBER staining of the tumors was not performed to confirm EBV presence within the tumor itself; however, we plan to do this in our future studies. Because of the small sample size of this study, we agree with the reviewer that a clear distinction between malaria-associated and cancer/EBV-driven immune remodeling is not possible at this stage, and only hypotheses can be generated. These hypotheses are guiding us as we set up a larger cohort.

Statistics: small sample size has been stated as a limitation in the discussion, **authors might want to exercise caution on the "correlation vs causation" front**.

*Response #19*: We have exercised more caution and now avoid implying causation in lines 364-365, and instead suggest as follows, “our results suggest that malaria, just like other inflammatory diseases, may be driving a shift in neutrophil subsets, and this may be lead to the accumulation of aged neutrophil subsets in circulation.”

Writing and Grammar: The manuscript will benefit from improved flow and language check to avoid making very strong claims that may not have fully supporting data, suggestive wording might be better. **Line 347 to 349 is incomplete.**

*Response #20*: We apologize for this oversight. In the revised manuscript,line 329-330 the sentence has been completed as follows, “To assess whether the observed neutrophil profiles were unique to BL, we compared them to children with other cancers (OC).” We also performed a language check to the entire manuscript.
